# Di-μ-chlorido-bis[chloridobis(dimethyl sulfoxide-κ*O*)tin(II)]

**DOI:** 10.1107/S1600536811009895

**Published:** 2011-03-26

**Authors:** Ioana Barbul, Richard A. Varga, Cristian Silvestru

**Affiliations:** a Faculty of Chemistry and Chemical Engineering, Babes-Bolyai University, Arany Janos Str. No. 11, RO-400028 Cluj-Napoca, Romania

## Abstract

The structure of the title compound, [Sn_2_Cl_4_(C_2_H_6_OS)_4_], contains dimers formed through weak Sn⋯Cl [3.691 (2) Å] inter­actions, resulting in a planar Sn_2_Cl_2_ core with an inversion center at the centre of the four-membered ring. The Sn^II^ atoms are penta­coordinated and have a distorted octa­hedral Ψ-SnCl_3_O_2_ coordination geometry. The O atoms from the dimethyl sulfoxide mol­ecules occupy *trans* positions, while the Cl atoms exhibit a meridional arrangement.

## Related literature

For related tin chlorides, see: Kisenyi *et al.* (1985 ▶); Kiriyama *et al.* (1973 ▶). For the structure of free DMSO, see: Viswamitra & Kannan (1966 ▶). 
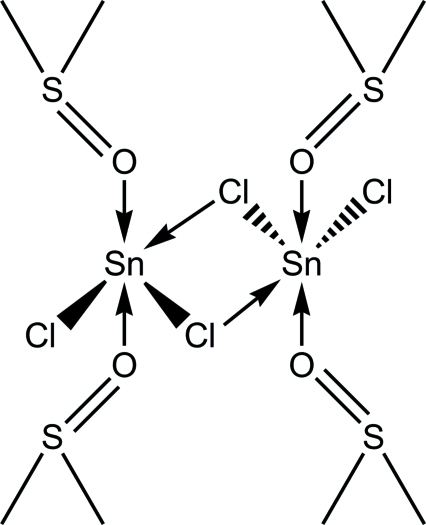


## Experimental

### 

#### Crystal data

[Sn_2_Cl_4_(C_2_H_6_OS)_4_]
*M* *_r_* = 691.70
Monoclinic, 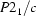

*a* = 11.1449 (17) Å
*b* = 13.349 (2) Å
*c* = 8.4394 (13) Å
β = 103.728 (2)°
*V* = 1219.7 (3) Å^3^
*Z* = 2
Mo *K*α radiation
μ = 2.84 mm^−1^
*T* = 297 K
0.28 × 0.25 × 0.23 mm

#### Data collection

Bruker SMART APEX CCD area-detector diffractometer
Absorption correction: multi-scan (*SADABS*; Bruker, 2000 ▶) *T*_min_ = 0.469, *T*_max_ = 0.523
8630 measured reflections
2148 independent reflections
1853 reflections with *I* > 2σ(*I*)
*R*_int_ = 0.062

#### Refinement

*R*[*F* ^2^ > 2σ(*F* ^2^)] = 0.050
*wR*(*F* ^2^) = 0.097
*S* = 1.18
2148 reflections
105 parameters
H-atom parameters constrained
Δρ_max_ = 0.57 e Å^−3^
Δρ_min_ = −0.72 e Å^−3^

### 

Data collection: *SMART* (Bruker, 2000 ▶); cell refinement: *SAINT-Plus* (Bruker, 2001 ▶); data reduction: *SAINT-Plus*; program(s) used to solve structure: *SHELXS97* (Sheldrick, 2008 ▶); program(s) used to refine structure: *SHELXL97* (Sheldrick, 2008 ▶); molecular graphics: *DIAMOND* (Brandenburg & Putz, 2006 ▶); software used to prepare material for publication: *publCIF* (Westrip, 2010 ▶).

## Supplementary Material

Crystal structure: contains datablocks I, global. DOI: 10.1107/S1600536811009895/vn2005sup1.cif

Structure factors: contains datablocks I. DOI: 10.1107/S1600536811009895/vn2005Isup2.hkl

Additional supplementary materials:  crystallographic information; 3D view; checkCIF report

## Figures and Tables

**Table:** 

Cl1—Sn1	2.4767 (19)
Cl2—Sn1	2.4886 (19)
O1—Sn1	2.382 (5)
O2—Sn1	2.371 (5)

**Table:** 

O2—Sn1—O1	166.36 (17)
O2—Sn1—Cl1	86.61 (13)
O1—Sn1—Cl1	85.99 (13)
O2—Sn1—Cl2	84.94 (14)
O1—Sn1—Cl2	84.15 (13)
Cl1—Sn1—Cl2	93.86 (7)

**Fig. 1.**
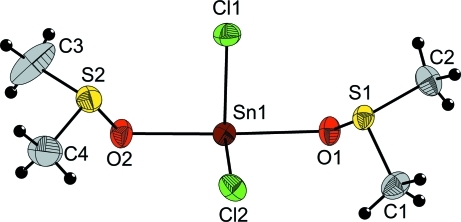
: View of the title compound showing the atom-numbering scheme. Displacement ellipsoids are drawn at the 30% probability level and H atoms as spheres of arbitrary radii.

**Fig. 2.**
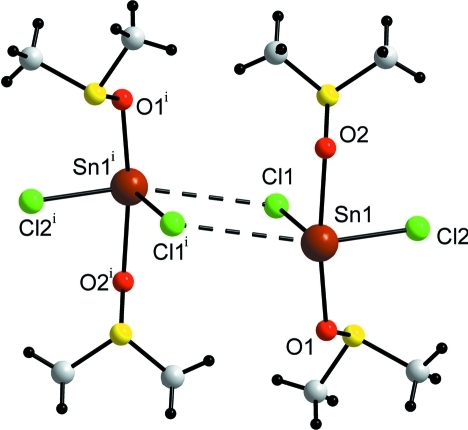
: Intermolecular interactions (represented with dashed lines) showing the formation of dimers in crystal structure of the title compound. Symmetry codes as in Table 1.

**Fig. 3.**
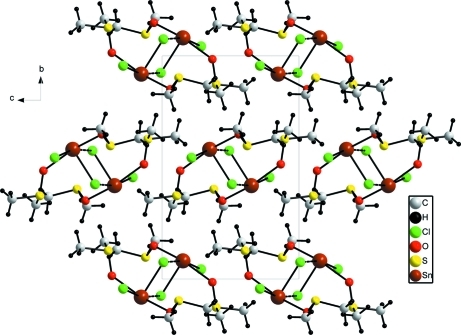
: Crystal packing of the title compound. View down the a axis.

## supplementary crystallographic information

### Comment

In an attempt to perform an oxidative addition of SnCl_2_ to an organic halide, the title compound was isolated as a by-product.

The tin(II) dichloride crystallizes with two dimethylsulfoxide molecules which coordinate to the metal center in a *trans* fashion through the oxygen atoms [O1—Sn1—O2 = 166.36 (17)°] (Figure 1). The molecular units are connected in dimers through weak Sn···Cl interactions [Sn1···Cl1^i^ = 3.691 (2) Å; symmetry code (i): -*x*, -*y* + 2, -*z*] *trans* to a Sn1—Cl2 bond [Cl2— Sn1···Cl1^i^ = 164.85 (6)°]. This results in a planar Sn_2_Cl_2_ core with an inversion centre in the middle of the four-membered ring (Figure 2). The chlorine bridges are asymmetric and the endocyclic angles around chlorine atoms [Sn1—Cl1—Sn1^i^ = 101.11 (5)°] are larger than the endocyclic angles around tin [Cl1—Sn1—Cl1^i^ = 78.90 (6)°].

In the dimer unit the tin atom is pentacoordinated in a distorted *pseudo*-octahedral coordination geometry, with the two chlorine atoms from the same molecular unit in *cis* positions [Cl1—Sn1—Cl2 = 93.86 (7)°] and a bridging chlorine atom *trans* to the free position. In contrast, in SnCl_4_.2DMSO (Kisenyi *et al.*, 1985) the tin atom is hexacoordinated, with the oxygen atoms from the dimethylsulfoxide in *cis* position, while the structure of SnCl_2_.2H_2_O is described as pyramidal (Kiriyama *et al.*, 1973) with only one water molecule bonded to the metal center.

The  Sn—O bond lengths (Table 1) are similar to those found in SnCl_2_.2H_2_O [2.331 (5) Å], but larger than in SnCl_4_.2DMSO [2.110 (9) and 2.110 (8) Å]. The Sn—Cl bonds follow the same pattern; those in SnCl_4_.2DMSO [range: 2.369 (3) - 2.406 (3) Å] are larger than in the title compound [Sn1—Cl1 = 2.4767 (19) Å, Sn1—Cl2 = 2.4886 (19) Å] and SnCl_2_.2H_2_O [2.500 (2) and 2.562 (2) Å]. This is consistent with the fact that SnCl_2_ is a weaker Lewis acid than SnCl_4_.

The S—O bonds [S1—O1 = 1.531 (5) Å, S2—O2 = 1.519 (5) Å] show a decrease of multiplicity from the S═O bond in the free ligand [S═O = 1.471 Å], due to the oxygen-tin interaction. The S—C bond lengths vary between 1.727 (11) and 1.779 (8) Å, which are similar with those from the free DMSO molecule (Viswamitra & Kannan, 1966).

In the strucure the dimers are stacked along the *a* axis and form layers stacking along the *b* axis, with alternate arrangement of the dimeric units in consecutive layers (Figure 3).

### Experimental

The title compound was isolated as a by-product after the workup of the reaction between SnCl_2_ to an organic halide performed in hot dimethyl sulfoxide (DMSO).

### Refinement

All hydrogen atoms were placed in calculated positions using a riding model, with C—H = 0.96 Å and with *U*_iso_= 1.5*U*_eq_ (C) for methyl H.

The data collection was done with 2 second irradiation time per frame over the complete sphere for a total data collection time of 2 hours. An earlier attempt to measure a crystal with a 10 second irradiation time per frame resulted in crystal decay after approximately 3 hours.

### Crystal data

**Table:**

[Sn_2_Cl_4_(C_2_H_6_OS)_4_]	*F*(000) = 672
*M**_r_* = 691.70	*D*_x_ = 1.883 Mg m^−^^3^
Monoclinic, *P*2_1_/*c*	Mo *K*α radiation, λ = 0.71073 Å
Hall symbol: -P 2ybc	Cell parameters from 3345 reflections
*a* = 11.1449 (17) Å	θ = 2.4–26.6°
*b* = 13.349 (2) Å	µ = 2.84 mm^−^^1^
*c* = 8.4394 (13) Å	*T* = 297 K
β = 103.728 (2)°	Block, colourless
*V* = 1219.7 (3) Å^3^	0.28 × 0.25 × 0.23 mm
*Z* = 2	

### Data collection

**Table:**

Bruker SMART APEX CCD area-detector diffractometer	2148 independent reflections
Radiation source: fine-focus sealed tube	1853 reflections with *I* > 2σ(*I*)
graphite	*R*_int_ = 0.062
φ and ω scans	θ_max_ = 25.0°, θ_min_ = 2.4°
Absorption correction: multi-scan (*SADABS*; Bruker, 2000)	*h* = −13→13
*T*_min_ = 0.469, *T*_max_ = 0.523	*k* = −15→15
8630 measured reflections	*l* = −10→10

### Refinement

**Table:**

Refinement on *F*^2^	Secondary atom site location: difference Fourier map
Least-squares matrix: full	Hydrogen site location: inferred from neighbouring sites
*R*[*F*^2^ > 2σ(*F*^2^)] = 0.050	H-atom parameters constrained
*wR*(*F*^2^) = 0.097	*w* = 1/[σ^2^(*F*_o_^2^) + (0.0208*P*)^2^ + 2.685*P*] where *P* = (*F*_o_^2^ + 2*F*_c_^2^)/3
*S* = 1.18	(Δ/σ)_max_ = 0.001
2148 reflections	Δρ_max_ = 0.57 e Å^−^^3^
105 parameters	Δρ_min_ = −0.71 e Å^−^^3^
0 restraints	Extinction correction: *SHELXL97* (Sheldrick, 2008), Fc^*^=kFc[1+0.001xFc^2^λ^3^/sin(2θ)]^-1/4^
Primary atom site location: structure-invariant direct methods	Extinction coefficient: 0.128 (4)

### Special details

**Table:**

Geometry. All e.s.d.'s (except the e.s.d. in the dihedral angle between two l.s. planes) are estimated using the full covariance matrix. The cell e.s.d.'s are taken into account individually in the estimation of e.s.d.'s in distances, angles and torsion angles; correlations between e.s.d.'s in cell parameters are only used when they are defined by crystal symmetry. An approximate (isotropic) treatment of cell e.s.d.'s is used for estimating e.s.d.'s involving l.s. planes.
Refinement. Refinement of *F*^2^ against ALL reflections. The weighted *R*-factor *wR* and goodness of fit *S* are based on *F*^2^, conventional *R*-factors *R* are based on *F*, with *F* set to zero for negative *F*^2^. The threshold expression of *F*^2^ > σ(*F*^2^) is used only for calculating *R*-factors(gt) *etc*. and is not relevant to the choice of reflections for refinement. *R*-factors based on *F*^2^ are statistically about twice as large as those based on *F*, and *R*- factors based on ALL data will be even larger.

### Fractional atomic coordinates and isotropic or equivalent isotropic displacement parameters (Å^2^)

**Table:**

	*x*	*y*	*z*	*U*_iso_*/*U*_eq_	
C1	0.5033 (7)	0.8276 (6)	−0.0501 (11)	0.068 (2)	
H1A	0.5258	0.8292	0.0670	0.102*	
H1B	0.5703	0.8529	−0.0918	0.102*	
H1C	0.4856	0.7598	−0.0864	0.102*	
C2	0.3399 (8)	0.8695 (7)	−0.3317 (9)	0.069 (2)	
H2A	0.3327	0.7980	−0.3425	0.103*	
H2B	0.4060	0.8924	−0.3775	0.103*	
H2C	0.2639	0.9002	−0.3886	0.103*	
C3	0.0141 (10)	1.1657 (9)	0.422 (2)	0.140 (6)	
H3A	−0.0474	1.1587	0.3220	0.211*	
H3B	0.0195	1.2346	0.4557	0.211*	
H3C	−0.0083	1.1254	0.5051	0.211*	
C4	0.2396 (11)	1.1408 (8)	0.5967 (12)	0.101 (4)	
H4A	0.1994	1.1045	0.6677	0.152*	
H4B	0.2439	1.2105	0.6254	0.152*	
H4C	0.3216	1.1150	0.6077	0.152*	
Cl1	0.1548 (2)	1.07531 (15)	−0.0095 (3)	0.0633 (5)	
Cl2	0.40441 (17)	0.95628 (17)	0.2850 (2)	0.0614 (6)	
O1	0.2681 (4)	0.8512 (4)	−0.0601 (6)	0.0534 (13)	
O2	0.1396 (5)	1.0145 (4)	0.3674 (6)	0.0600 (14)	
S1	0.37123 (18)	0.90252 (13)	−0.1217 (2)	0.0469 (5)	
S2	0.1554 (2)	1.12668 (15)	0.3945 (3)	0.0617 (6)	
Sn1	0.18486 (4)	0.91864 (3)	0.15234 (6)	0.0448 (3)	

### Atomic displacement parameters (Å^2^)

**Table:**

	*U*^11^	*U*^22^	*U*^33^	*U*^12^	*U*^13^	*U*^23^
C1	0.054 (5)	0.064 (5)	0.090 (6)	0.006 (4)	0.025 (5)	0.007 (5)
C2	0.079 (6)	0.073 (6)	0.055 (5)	−0.015 (5)	0.018 (4)	−0.008 (4)
C3	0.074 (8)	0.075 (7)	0.267 (19)	0.011 (6)	0.029 (9)	−0.027 (9)
C4	0.139 (10)	0.070 (7)	0.080 (7)	0.004 (6)	−0.003 (7)	−0.012 (5)
Cl1	0.0684 (13)	0.0577 (12)	0.0648 (12)	0.0102 (10)	0.0178 (10)	0.0151 (10)
Cl2	0.0490 (11)	0.0818 (14)	0.0511 (11)	−0.0116 (10)	0.0073 (9)	0.0026 (10)
O1	0.056 (3)	0.053 (3)	0.059 (3)	−0.009 (2)	0.028 (3)	−0.010 (2)
O2	0.079 (4)	0.045 (3)	0.062 (3)	−0.004 (3)	0.030 (3)	−0.011 (2)
S1	0.0549 (11)	0.0371 (10)	0.0520 (11)	−0.0037 (8)	0.0194 (9)	−0.0055 (8)
S2	0.0799 (15)	0.0487 (12)	0.0594 (13)	−0.0044 (10)	0.0225 (11)	0.0024 (9)
Sn1	0.0439 (4)	0.0407 (3)	0.0514 (4)	−0.0045 (2)	0.0147 (2)	0.0007 (2)

### Geometric parameters (Å, °)

**Table:**

C1—S1	1.764 (8)	C3—H3C	0.9600
C1—H1A	0.9600	C4—S2	1.751 (10)
C1—H1B	0.9600	C4—H4A	0.9600
C1—H1C	0.9600	C4—H4B	0.9600
C2—S1	1.779 (8)	C4—H4C	0.9600
C2—H2A	0.9600	Cl1—Sn1	2.4767 (19)
C2—H2B	0.9600	Cl2—Sn1	2.4886 (19)
C2—H2C	0.9600	O1—S1	1.531 (5)
C3—S2	1.727 (11)	O1—Sn1	2.382 (5)
C3—H3A	0.9600	O2—S2	1.519 (5)
C3—H3B	0.9600	O2—Sn1	2.371 (5)
			
S1—C1—H1A	109.5	S2—C4—H4C	109.5
S1—C1—H1B	109.5	H4A—C4—H4C	109.5
H1A—C1—H1B	109.5	H4B—C4—H4C	109.5
S1—C1—H1C	109.5	S1—O1—Sn1	123.0 (3)
H1A—C1—H1C	109.5	S2—O2—Sn1	127.5 (3)
H1B—C1—H1C	109.5	O1—S1—C1	105.2 (4)
S1—C2—H2A	109.5	O1—S1—C2	104.0 (3)
S1—C2—H2B	109.5	C1—S1—C2	98.7 (4)
H2A—C2—H2B	109.5	O2—S2—C3	103.9 (5)
S1—C2—H2C	109.5	O2—S2—C4	105.6 (4)
H2A—C2—H2C	109.5	C3—S2—C4	97.4 (7)
H2B—C2—H2C	109.5	O2—Sn1—O1	166.36 (17)
S2—C3—H3A	109.5	O2—Sn1—Cl1	86.61 (13)
S2—C3—H3B	109.5	O1—Sn1—Cl1	85.99 (13)
H3A—C3—H3B	109.5	O2—Sn1—Cl2	84.94 (14)
S2—C3—H3C	109.5	O1—Sn1—Cl2	84.15 (13)
H3A—C3—H3C	109.5	Cl1—Sn1—Cl2	93.86 (7)
H3B—C3—H3C	109.5	Sn1—Cl1—Sn1^i^	101.11 (5)
S2—C4—H4A	109.5	Cl1—Sn1—Cl1^i^	78.90 (6)
S2—C4—H4B	109.5	Cl2—Sn1—Cl1^i^	164.85 (6)
H4A—C4—H4B	109.5		
			
Sn1—O1—S1—C1	108.4 (4)	S2—O2—Sn1—Cl1	−24.9 (4)
Sn1—O1—S1—C2	−148.3 (4)	S2—O2—Sn1—Cl2	69.2 (4)
Sn1—O2—S2—C3	129.3 (7)	S1—O1—Sn1—O2	−5.1 (10)
Sn1—O2—S2—C4	−128.7 (5)	S1—O1—Sn1—Cl1	52.2 (3)
S2—O2—Sn1—O1	32.3 (10)	S1—O1—Sn1—Cl2	−42.1 (3)

Symmetry codes: (i) −*x*, −*y*+2, −*z*.
